# Endometriosis in transgender men: recognizing the missing pieces

**DOI:** 10.3389/fmed.2023.1266131

**Published:** 2023-08-31

**Authors:** Alexandre Vallée, Anis Feki, Jean-Marc Ayoubi

**Affiliations:** ^1^Department of Epidemiology and Public Health, Foch Hospital, Suresnes, France; ^2^Department of Gynecology and Obstetrics, University Hospital of Fribourg, Fribourg, Switzerland; ^3^Department of Obstetrics, Gynecology and Reproductive Medicine, Foch Hospital, Suresnes, France; ^4^Medical School, University of Versailles, Saint-Quentin-en-Yvelines (UVSQ), Versailles, France

**Keywords:** transgender, endometriosis, healthcare, diagnosis, hormones

## Abstract

Endometriosis, traditionally associated with cisgender women, should be recognized as a significant issue for transgender men. This perspective highlights the need to address the unique experiences and challenges faced by transgender men with endometriosis. Diagnostic difficulties arise due to hormone therapy and surgical interventions, which can alter symptoms. Limited research in transgender men undergoing hysterectomy further complicates the understanding of endometriosis in this population. Healthcare providers must be aware of these challenges and adapt the diagnostic approaches accordingly. Education and inclusive care are essential to ensure timely and appropriate management of endometriosis in transgender men, ultimately improving their quality of life.

## Introduction

**Figure 1:**
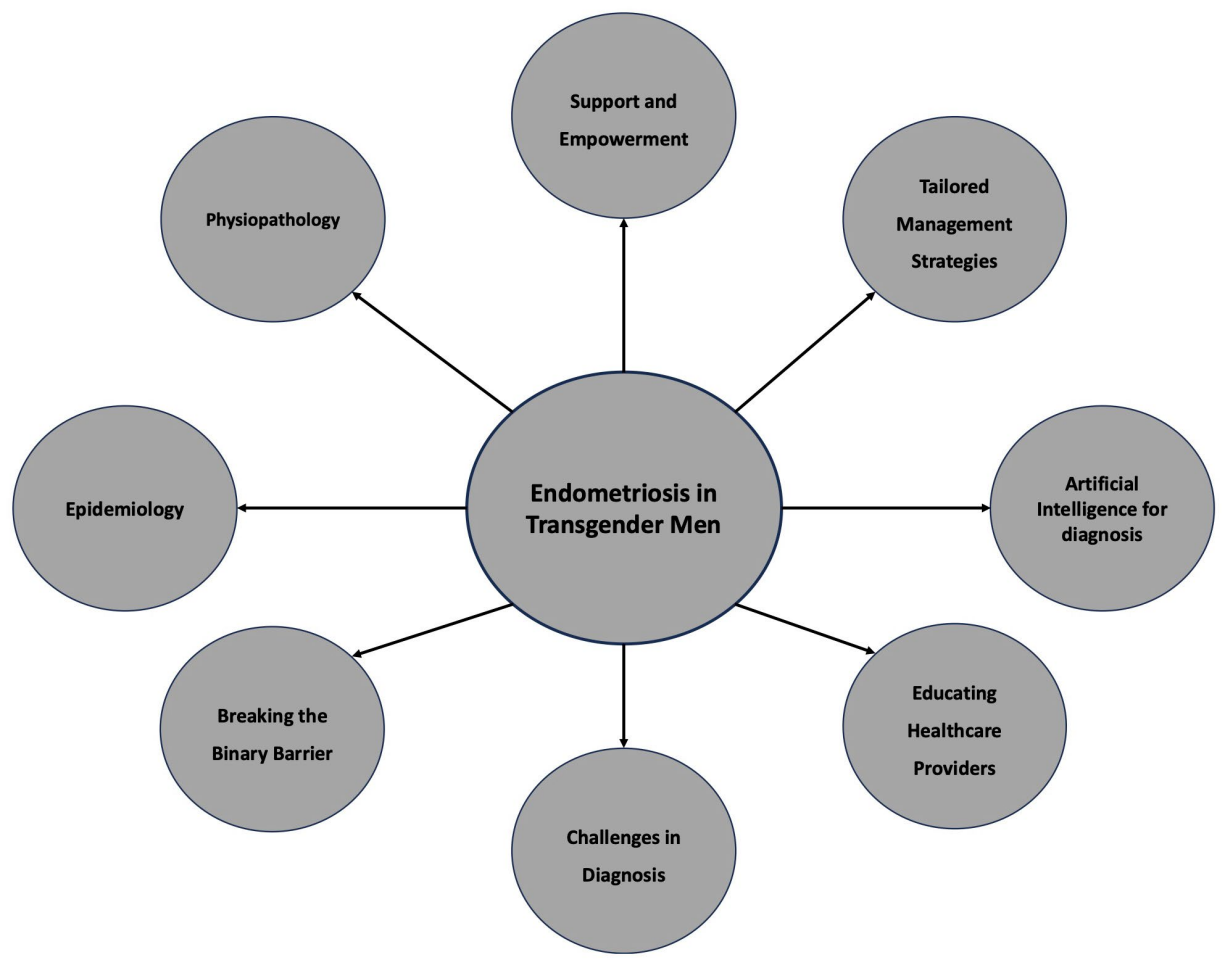
Raise awareness about endometriosis in transgender men.

Endometriosis is a complex and debilitating condition that has long been associated with cisgender women (1). However, as we strive for inclusivity in healthcare, it is crucial to shed light on the experiences of transgender men, who also face the challenges of endometriosis. Few studies have provided the prevalence of endometriosis among transgender men. However, the pooled prevalence of endometriosis could be estimated at 25.14, 95% CI (17.24%–33.94%) and the frequency of patients using testosterone without other medications and presenting dysmenorrhea was 70.58, 95% CI (63.87%–80.91%) (2–4). Furthermore, stage 1 (40%) and 2 (32%) endometriosis were the most reported findings (2). Among transgender men who underwent hysterectomy, 89.5% were on testosterone, 59.7% were amenorrheic, 43.2% had dysmenorrhea, 17.9% reported heavy menses, and 14.9% had irregular menses, 50.7% complained of pelvic pain (35.3% constant, 64.7% cyclic) (4). Moreover, endometriosis was found in 32% of patients who reported pelvic pain at the preoperative consultation and in 22% of patients who did not complain of pain (4, 5). This perspective aims to raise awareness about endometriosis in transgender men, emphasizing the need for research, education, and comprehensive care tailored to their unique circumstances (Figure 1).

## Physiopathology of endometriosis among transgender men

Only a limited number of studies have investigated pelvic organ pathology among transgender men who undergo hysterectomy, and the existing reports present conflicting results. For instance, Grimstad et al. (6) examined uterine pathology in transgender men undergoing hysterectomy as part of their gender affirmation process while on testosterone. Interestingly, most of the pathology reports from these individuals displayed active endometrial tissue. In contrast, Khalifa et al. (7) studied a similar group of patients and found that most specimens they assessed exhibited endometrial changes consistent with inactive endometrium. These mixed findings make it difficult to definitively determine the impact of testosterone on the endometrium. However, the presence of active endometrium in some patients suggests that complete cessation of ovarian function and/or endometrial activity might not occur for all individuals on testosterone.

These contrasting results could potentially be linked to the conversion of exogenous testosterone to estradiol in peripheral tissues, a process known as aromatization (1). While there are no studies specifically investigating trends in estradiol levels among transgender individuals on long-term testosterone therapy, it is plausible that elevated androgen levels could be transformed into estrogen in this clinical context, potentially resulting in a state of heightened estrogenic activity (1). As endometriosis is regarded as an estrogen-driven condition, this could contribute to its development. Although this hypothesis does not elucidate the precise mechanism underlying the increased occurrence of endometriosis in transgender individuals compared to their cisgender counterparts, it does explain their potential symptomatic presentation of the condition, even in cases where menstruation has ceased (1). To date, the risk of endometrial disease in transgender men using testosterone is unclear, and expert opinion recommendations for routine endometrial surveillance (ultrasound or biopsy) or primary surgical prevention (hysterectomy) of endometrial pathology are not evidence based (5, 8). The effect of gender-affirming hormone therapy with testosterone therapy on the endometrium is incompletely characterized, and the etiology of this distribution of endometrial findings is unknown (5). Moreover, the proliferative endometrium in transgender men could be explained by the persistent elevated serum estrogen observed in patients who retain their ovaries while using testosterone (9).

## Breaking the binary barrier

Transgender men assigned female at birth but identifying as male often find themselves navigating a healthcare system that fails to adequately address their specific needs (10). Endometriosis, primarily viewed as a “women’s issue”, is a prime example of this oversight (3). By recognizing and studying endometriosis in transgender men, we can dismantle the binary understanding of this condition and pave the way for more inclusive healthcare practices.

## Challenges in diagnosis

Diagnosing endometriosis in transgender men presents unique challenges. Given their hormone therapy (testosterone) and potential surgical interventions such as hysterectomy and oophorectomy, the symptoms and manifestations of endometriosis may differ from those experienced by cisgender women (11). Healthcare providers must be vigilant in considering endometriosis as a potential cause of pelvic pain, even in transgender men, and adapt the diagnostic approaches accordingly (4).

Endometriosis is often perceived as a condition exclusive to cisgender women (1). This limited understanding can result in healthcare providers overlooking endometriosis as a potential diagnosis in transgender men. It is essential to raise awareness and to educate healthcare professionals about the possibility of endometriosis in this population.

Testosterone therapy, which is commonly used during gender transition, can influence the symptoms and presentation of endometriosis in transgender men (12). The hormonal changes brought about by testosterone can mask or alter typical symptoms, such as changes in menstrual patterns or pelvic pain (13). Healthcare providers must be knowledgeable about these potential variations to ensure accurate diagnosis.

There is a scarcity of studies examining pelvic organ pathology in transgender men undergoing hysterectomy, and the existing reports are limited and present controversial findings (14). A previous study examined the characteristics of uterine pathology in 94 transgender men receiving testosterone treatment who underwent hysterectomy as part of their gender affirmation process (6). Interestingly, most of the pathology reports indicated the presence of an active endometrium in these patients. In contrast, other studies have investigated similar groups of patients and reported that most of the evaluated specimens showed endometrial changes consistent with an inactive endometrium (7, 15). These mixed findings make it challenging to definitively determine the effects of testosterone on the endometrium. However, considering the report of an active endometrium in some patients, it can be inferred that certain individuals do not experience complete cessation of ovarian function and/or endometrial activity while on testosterone therapy. This implies that transgender men predisposed to endometriosis may still have active disease, even when undergoing testosterone treatment.

Due to the lack of awareness and altered symptoms, there can be delays in diagnosing endometriosis in transgender men. Patients may experience dismissive attitudes or have their symptoms attributed to other causes, leading to a prolonged period of suffering and reduced quality of life. Overcoming diagnostic delays requires a proactive and open-minded approach from healthcare providers (16).

There is a dearth of research specifically focused on endometriosis in transgender men. The absence of comprehensive guidelines and evidence-based practices tailored to this population further hinders accurate diagnosis. More research is needed to understand the prevalence, pathophysiology, and optimal diagnostic approaches for endometriosis in transgender men. Traditional diagnostic modalities, such as imaging like ultrasound or magnetic resonance imaging (MRI), may not provide definitive results to diagnose endometriosis in transgender men (17). These imaging techniques may not effectively capture the presence of endometrial lesions in neovaginas or residual endometrial tissue in transgender men who have undergone hysterectomy (17). Consequently, a more nuanced approach could be use, such as laparoscopy or specialized imaging techniques [MRI using artificial intelligence (18)] for accurate diagnosis. AI models using biomarkers could be accurate with investigations focused on protein ratios (19), metabolites (20) and miRNAs (21). Moreover, other predictive models could use protein spectra (22) in association with neural networks algorithms (23), and large protein-coding gene datasets from transcriptomics and methylomics data coupled with machine learning models (24, 25). Healthcare providers must approach the diagnostic process with sensitivity and open communication. Creating a safe and inclusive environment enables transgender men to discuss their symptoms openly, allowing for a more accurate assessment (26). Healthcare providers should proactively inquire about the gender transition history, hormonal therapies, and any complications related to gender-affirming surgeries that may contribute to endometriosis-like symptoms.

## Educating healthcare providers

Healthcare providers must receive adequate training and education on transgender healthcare and endometriosis management. Many medical professionals may lack knowledge in both areas, resulting in misdiagnoses, delayed interventions, or the dismissal of symptoms (27). By bridging this educational gap, we can ensure that transgender men receive competent and compassionate care from healthcare providers who understand the nuanced intersection of their gender identity and endometriosis.

## Tailored management strategies

Effective management of endometriosis in transgender men requires tailored approaches (1). Hormone therapy, the cornerstone of gender transition, may have an impact on the growth and symptoms of endometriosis (28). Healthcare providers should be knowledgeable about the potential interactions between testosterone therapy and endometriosis, ensuring that treatment plans strike a balance between gender-affirming care and mitigating endometriosis-related symptoms. The prevalence of endometriosis in transgender men is higher than the female cisgender population (2). Thus, surgeons should perform a careful intraoperative assessment of endometriotic foci within transgender men. But, to date, very few data are available and future prospective studies are needed.

Furthermore, a comprehensive biopsychosocial approach that encompasses various factors contributing to everyone’s situation is essential. This approach may involve medical treatments, addressing sexual function, dealing with pain hypersensitivity, and considering psychological aspects like post-traumatic stress disorder (29).

A significant number of transgender individuals seek hysterectomy and/or oophorectomy as part of their gender affirmation process or due to persistent pelvic pain or abnormal bleeding. Among those who underwent hysterectomy, 72% reported experiencing relief from pelvic pain symptoms following the procedure (13). This surgery effectively stops ongoing menstruation, which is particularly prevalent in those experiencing pain after beginning testosterone therapy.

Although further research is required to explore the potential link between elevated pelvic floor muscle tension and pelvic pain in transgender individuals undergoing testosterone therapy for gender affirmation, a recent systematic review of pelvic floor physical therapy aimed at releasing myofascial trigger points demonstrated positive and beneficial outcomes, especially for individuals dealing with chronic pelvic pain and dyspareunia (30). Considering the limited available options to alleviate often incapacitating pelvic pain in transgender individuals undergoing testosterone therapy, pelvic floor physical therapy emerges as a viable and low-risk treatment strategy (29). A program focused on reducing pelvic floor muscle tension, emphasizing improved muscle function quality and the relaxation phase of contractions, holds promise in this clinical setting.

## Support and empowerment

Support networks and advocacy groups play a crucial role in empowering transgender men with endometriosis. By fostering a sense of community, raising awareness, and providing access to resources, these organizations can offer the much-needed support that helps transgender men navigate the challenges of endometriosis. Moreover, incorporating the voices of transgender men in policymaking and healthcare guidelines will ensure that their unique experiences and needs are considered (31).

## Conclusion

Endometriosis does not discriminate based on gender identity, and it is high time we recognized the existence and impact of this condition on transgender men. By promoting research, education, and comprehensive care, we can break down the barriers that hinder effective diagnosis and management of endometriosis in this marginalized population. It is our responsibility as healthcare providers, researchers, and advocates to address this gap in understanding and to provide equitable healthcare for all individuals, irrespective of their gender identity.

## Data availability statement

The original contributions presented in the study are included in the article/supplementary material, further inquiries can be directed to the corresponding author.

## Author contributions

AV: Conceptualization, Validation, Writing – original draft, Writing – review & editing. AF: Validation, Writing – original draft, Writing – review & editing. J-MA: Validation, Writing – original draft, Writing – review & editing.

## Funding

The author(s) declare that no financial support was received for the research, authorship, and/or publication of this article.

## Conflict of interest

The authors declare that the research was conducted in the absence of any commercial or financial relationships that could be construed as a potential conflict of interest.

The author(s) declared that they were an editorial board member of Frontiers, at the time of submission. This had no impact on the peer review process and the final decision.

## Publisher’s note

All claims expressed in this article are solely those of the authors and do not necessarily represent those of their affiliated organizations, or those of the publisher, the editors and the reviewers. Any product that may be evaluated in this article, or claim that may be made by its manufacturer, is not guaranteed or endorsed by the publisher.
